# The internal structure of transformational leadership as perceived by clinical nurses: a network analysis

**DOI:** 10.3389/fpsyg.2026.1878851

**Published:** 2026-07-29

**Authors:** Ning Zha, Haojun Hu, Kai Zhang, Changchang Chen, Huan Xu, Chao Wu, Hongjuan Lang

**Affiliations:** 1 Nursing Department, Air Force Medical University, Xi’an, Shaanxi, China; 2 Medical Clinic of the Air Force Unit Hospital, Wuhan, China; 3 Shaanxi University of Chinese Medicine, Xianyang, China; 4 Field and Disaster Nursing Teaching and Research Section, School of Nursing, Air Force Medical University, Xi’an, Shaanxi, China

**Keywords:** clinical nurse, internal structure, leadership perception, network analysis, nursing management, transformational leadership

## Abstract

**Introduction:**

Transformational leadership, a proactive leadership style, is positively associated with increased nurses’ job satisfaction, reduced occupational burnout, and enhanced retention intention. However, existing research has predominantly relied on conventional statistical methods, whereas network analysis—which examines the intrinsic connections among the dimensions of transformational leadership—remains underutilized. This study used network analysis to explore the interrelationships among dimensions of transformational leadership in clinical nursing. It addressed the research gap left by conventional statistical methods and provided empirical evidence to optimize nursing leadership practices and enhance clinical team management efficacy.

**Methods:**

From October to December 2025, we recruited 948 clinical nurses from 10 hospitals using a convenience sampling method. A total of 836 nurses provided valid responses. Transformational leadership was assessed using the Transformational Leadership Questionnaire. The collected data were analyzed using network analysis.

**Results:**

Within the overall network structure, nodes D6 (“Demonstrate decisiveness and assertiveness in management, and be adept at resolving complex issues”), A7 (“Be willing to share both hardships and successes with the team”), C5 (“Show concern for team members’ professional work, personal wellbeing, and career growth, and offer sincere developmental advice”), and C6 (“Focus on creating opportunities for team members to leverage their strengths”) exhibit the highest strength and expected influence. Meanwhile, nodes C1 (“Consider individual circumstances when interacting with team members”), B6 (“Regularly collaborate with the team to analyze how your work aligns with and impacts departmental and hospital objectives”), B1 (“Facilitate an understanding of the hospital and department’s developmental prospects”), and D1 (“Possess exceptional professional competence”) are the core nodes with the highest bridge expected influence.

**Conclusion:**

Network analysis revealed that transformational leadership in clinical nursing constitutes a complex, interconnected network. In nursing manager training, core nodes (D6, A7, C5, C6) and bridge nodes (C1, B6, B1, D1) may be prioritized to potentially enhance leadership competency, align nurses’ career goals with hospital objectives, and foster synergistic leadership interactions.

## 1 Introduction

According to the 2025 State of the World’s Nursing Report, the global nursing shortage was 5.8 million in 2023 and is projected to decline to 4.1 million by 2030. The nursing profession faces multiple challenges, including insufficient educational resources, suboptimal working conditions, and weak leadership (World Health Organization, 2025). The persistent effects of the COVID−19 pandemic have further worsened working conditions for practicing nurses and exacerbated the global nursing shortage in the post-pandemic period (Buckley et al., 2025). Against this backdrop, fostering a positive nursing practice environment and strengthening nursing leadership have become key strategies to alleviate burnout among nurses, reduce turnover, and address staffing shortages. Research (Page and Carter, 2026) indicates that leaders play a crucial role in fostering and maintaining a positive nursing practice environment. Effective leadership behaviors, including clear communication, a visible and approachable frontline presence, recognition of nurses’ value, and platforms for professional development, can foster a high-quality nursing practice environment. Consequently, these behaviors enhance nurses’ professional wellbeing and job satisfaction (Page and Carter, 2026). Research indicates that the core traits of nurse leaders include professional expertise, visibility, empathy, approachability, flexible support, active listening, and foresight. In recent decades, nurse leaders have been instrumental in driving the shift of nursing leadership models toward transformational leadership (Kvist, 2026). Studies have also identified three prevalent leadership styles in nursing: transactional, laissez-faire, and transformational (Chen et al., 2024).

Transformational leadership was initially conceptualized by Burns in the 1970s and later expanded by Bass and Avolio (1992). This framework identifies four core leadership behaviors: idealized influence, inspirational motivation, intellectual stimulation, and individualized consideration. Idealized influence is characterized by subordinates’ deep trust in their leader and alignment with the leader’s values. Such leaders earn the team’s highest respect and consistently embody the values and behavioral standards they advocate through word and action. Inspirational motivation fosters a team spirit marked by positivity, appeal, and enthusiasm. Its core involves imbuing work with value and meaning, promoting followers’ growth through challenging tasks, and igniting their forward-looking vision and motivation to achieve goals. Intellectual stimulation emphasizes cultivating team members’ innovative thinking and creativity. It encourages managers to challenge existing work paradigms and assumptions, critically examine current practices, and explore new approaches. Individualized consideration involves managers acting as mentors who provide tailored guidance to support followers’ growth (Bass and Avolio, 1992). Transformational managers focus on followers’ individual needs, development, and growth potential by offering customized empowerment (Bass et al., 2003). Transformational leadership positively impacts employee satisfaction, engagement, and performance (Gebreheat et al., 2023). It is also a prevalent contemporary leadership style among nursing managers (Haoyan et al., 2023). A recent review (Hult et al., 2023) concluded that relational leadership styles, particularly transformational leadership, are positively correlated with improvements in standard nursing outcomes, such as lower adverse event rates, enhanced nurse performance, and higher patient satisfaction. By advocating for and empowering nurses, transformational leadership helps ensure that patients receive high-quality, safe nursing care. This goal remains central to the development of the nursing discipline and the cultivation of nursing leadership (Nurmeksela et al., 2025).

However, prior research has largely relied on mediation and regression analyses to investigate the links between transformational leadership and related variables. These approaches often rely on aggregated scale scores and assume independence among variables, thus failing to account for potential interaction effects across or within dimensions (Alhusban et al., 2026; Wu L. et al., 2025). Traditional statistical methods have notable limitations. On one hand, they often cannot accurately determine the relative importance of nodes in network structures, thereby failing to clarify key distinctions among elements. On the other hand, as the number of variables increases, the likelihood of spurious correlations among them rises substantially (Wu et al., 2023; Ye et al., 2025). Consequently, these limitations hinder a clear understanding of the interaction mechanisms among transformational leadership measurement items.

Network analysis can effectively overcome these limitations (Yang et al., 2025). This method uses partial correlation analysis to control for confounding factors, thereby accurately identifying direct relationships both among transformational leadership dimensions and across different items. Though applying regularization constraints to induce network sparsity, the method effectively removes spurious correlations and improves model stability in high-dimensional datasets. By computing node centrality metrics, this approach quantifies the influence of individual dimensions or items within the overall network, thereby providing more targeted empirical evidence for analyzing the internal mechanisms of transformational leadership (Hevey, 2018).

Given the limitations of existing research and the advantages of network analysis, this study employs network analysis to systematically examine the interaction mechanisms among the internal dimensions and measurement items of transformational leadership in nursing. This approach aims to identify core elements and key causal pathways. Based on these findings, the study will offer targeted practical insights for shaping leadership styles and cultivating transformational leadership competencies in nursing. Thus, it will expand the application and development of transformational leadership theory in nursing management.

## 2 Materials and methods

### 2.1 Participants

From October to December 2025, a cross-sectional survey was conducted among 948 nurses recruited from 10 hospitals. Participants were recruited from hospitals in Beijing, Jiangsu, Shaanxi, Henan, and Sichuan provinces, China. A convenience sampling method was used for participant selection. The inclusion criteria were as follows: (1) possession of a valid Chinese nursing license; (2) at least 1 year of clinical experience; (3) provision of informed consent and voluntary participation. Exclusion criteria were: (1) interns or rotating nurses; (2) nurses on leave or absent from duty during the survey period. The baseline characteristics of the participants, derived from sampling calculations, are summarized in Table 1.

**Table 1:** Description and univariate analysis of transformational leadership among clinical nurses (*N* = 836).

Variable	Category	*N*	Percentage (%)	Transformational leadership *M* ± SD	*t*/*F*	*p*
Gender	Male	46	0.06	107.46 ± 16.32	−1.18^a^	0.25
	Female	790	0.94	110.33 ± 12.27		
Age (years)	≤25	64	0.08	108.61 ± 13.38	1.40^b^	0.24
	26–35	326	0.39	110.11 ± 12.92		
	36–45	389	0.47	110.84 ± 12.09		
	>45	57	0.07	107.77 ± 12.14		
Working years (years)	≤5	162	0.19	109.58 ± 13.00	1.13^b^	0.34
	6–10	134	0.16	110.26 ± 13.47		
	11–15	249	0.30	109.68 ± 12.32		
	16–20	156	0.19	112.04 ± 11.76		
	>20	135	0.16	109.56 ± 12.24		
Professional title	Nurse or senior nurse	300	0.36	110.00 ± 13.42	0.05^b^	0.96
	Supervising nurse	450	0.54	110.27 ± 12.31		
	Associate chief nurse and above	86	0.10	110.28 ± 10.46		
Education level	Junior college	67	0.08	108.58 ± 15.76	0.14^b^	0.55
	Undergraduate	751	0.90	110.30 ± 12.26		
	Master’s degree or higher	18	0.02	110.67 ± 10.45		
Department	Infectious diseases department	67	0.08	111.60 ± 11.61	0.42^b^	0.79
	Internal medicine department	307	0.37	110.34 ± 12.55		
	Surgery department	232	0.28	110.21 ± 12.19		
	Emergency department	115	0.14	109.80 ± 12.38		
	Intensive care unit	115	0.14	109.22 ± 13.89		
Marital status	Unmarried	173	0.21	109.62 ± 13.71	0.48^b^	0.62
	Married	646	0.77	110.38 ± 12.21		
	divorced or widowed	17	0.02	108.12 ± 12.76		

^a^*t*-test; ^b^ANOVA.

### 2.2 Sample size calculation

The threshold parameter represents the critical value for each node, calculated as *n* = *N*, where *N* is the number of nodes in the network. The pairwise association parameter reflects the strength of association between node pairs, calculated as *n* = *N* (*N* − 1)/2. Thus, the required sample size is given by: Sample size = Threshold parameter + Pairwise association parameter. In this study, we used the 26-item Transformational Leadership Questionnaire developed by Li and Shi (2005). To account for a possible 20% non-response rate (Wu et al., 2020), the minimum sample size was increased to 422. Finally, 948 clinical nurses were enrolled in the study.

### 2.3 Data collection

After obtaining hospital administration approval, trained investigators distributed questionnaires to eligible nurses and provided a detailed explanation of the study’s objectives and content. Upon obtaining electronic informed consent, the nurses were asked to complete the online questionnaires independently and anonymously using QuestionStar. To ensure data quality, several control measures were implemented: restricting one response per IP address, excluding questionnaires completed in less than 2 min, and discarding incomplete or clearly patterned responses. In total, 948 questionnaires were collected. Following quality review, 836 valid responses were retained, resulting in an effective response rate of 88.19%.

### 2.4 Measurement

#### 2.4.1 Demographic information

The collected demographic data comprised age, gender, years of service, professional title, educational background, departmental affiliation, and marital status.

#### 2.4.2 Transformational leadership

Transformational leadership was assessed using the Transformational Leadership Questionnaire developed by Li and Shi (2005). This questionnaire was adapted from Bass and Avolio’s original transformational leadership framework to better align with Chinese cultural and organizational contexts (Li and Shi, 2005). The questionnaire comprises four dimensions: Moral Modeling (8 items), Inspirational Motivation (6 items), Individual Consideration (6 items), and Charisma (6 items). Items were rated on a 5-point Likert scale ranging from 1 (“strongly disagree”) to 5 (“strongly agree”), yielding total scores between 26 and 130. Higher scores reflect stronger perceived transformational leadership among nurses. This questionnaire has demonstrated high reliability and validity in Chinese cultural settings (Huang et al., 2025). In this study, the overall Cronbach’s alpha coefficient for the questionnaire was 0.946. The Cronbach’s alpha coefficient for the four dimensions was from 0.802 to 0.864. All values exceeded 0.80, further supporting the scale’s internal consistency reliability.

### 2.5 Data analysis

Data analysis was performed using SPSS 27.0 and RStudio (version 2025.09.1+401). Descriptive analyses, including sociodemographic characteristics and transformational leadership scores, were conducted with SPSS. The normality of data distribution was assessed with the Shapiro–Wilk test. Continuous data with a normal distribution are expressed as mean (*M*) and standard deviation (SD); categorical data are summarized as frequencies and percentages. Differences in scores across groups were compared using one-way analysis of variance (ANOVA). The network model was constructed using RStudio. The key analytical steps comprised: (1) estimating and visualizing the symptom network; (2) calculating network centrality indices and predictability; (3) assessing network accuracy and stability; and (4) estimating bridge centrality to identify bridge items that connect the four dimensions of transformational leadership.

#### 2.5.1 Symptom network estimation

Network analysis was conducted in RStudio. The “bootnet” and “qgraph” packages (Epskamp et al., 2012; Epskamp and Fried, 2018) were used to construct and visualize the network structure. The R package “qgraph” was employed to examine the relationships between items of the transformational leadership dimensions. First, a Gaussian Graph Model (GGM) was applied to estimate the network structure. Second, the least absolute shrinkage and selection operator (LASSO) regularization was implemented alongside the Extended Bayesian Information Criterion (EBIC) to simplify the network. This approach shrinks trivial correlations to zero, thereby reducing spurious connections (Yang et al., 2025). The EBIC hyperparameter was set to 0.50.

Nodes represent different aspects of transformational leadership, and edges denote the connections between them. In this network, blue edges represent positive correlations, red edges represent negative correlations, and thicker edges indicate stronger associations (Epskamp et al., 2012). The network comprises 8 nodes linked to moral modeling, and 6 nodes each associated with inspirational motivation, individual consideration, and charisma. We used the “NetworkComparisonTest” package to test for significant differences in node strength and edge weight (Wang et al., 2025).

#### 2.5.2 Centrality and predictability

A node’s centrality measures its direct connections with other nodes. Using the “centralityPlot” function from the R package “qgraph,” we computed four centrality indices—node strength, closeness, betweenness, and expected influence—to assess the relative importance of each node in the network (Chen et al., 2025). Node strength reflects the intensity of a node’s direct connections; closeness measures how efficiently a node is connected to all other nodes indirectly; betweenness indicates a node’s role in facilitating connections along the shortest paths between other nodes; expected influence estimates a node’s overall impact on the network (Zheng et al., 2024). Previous research suggests that node strength is more relevant to psychological constructs and demonstrates greater stability than closeness or betweenness centrality (McNally, 2016; Epskamp et al., 2018).

Simultaneously, we used the “bridge” function from the R package “networktools” to compute bridge strength and bridge expected influence (Opsahl et al., 2010). Bridge strength and bridge expected influence both quantify a node’s total connectivity to nodes outside its own community (Jones et al., 2021). Bridge strength is defined as the sum of the absolute values of a node’s connections to other communities; bridge expected influence serves as a network stability metric, reflecting the disruption in overall connectivity if the node is removed (Jones et al., 2021). Two methods are available for calculating bridge expected influence: Bridge expected influence (1-step) and Bridge expected influence (2-step) (Liu et al., 2024). Based on prior research, we incorporated the one-step bridge expected influence in our analysis (Jin et al., 2022).

Previous studies show that in networks with coexisting positive and negative correlations, expected influence and bridging expected influence are more representative than traditional centrality measures like strength and bridge strength (Tang et al., 2024). A node with higher expected influence plays a more significant role in network dynamics (Robinaugh et al., 2016). Higher bridging expected influence of a node signifies a greater risk that effects from that node will propagate across communities (Li et al., 2025). Following prior recommendations, this study reports centrality indices for the top four nodes (Bringmann et al., 2019).

#### 2.5.3 Accuracy and stability of the network

To evaluate the robustness of the network, we assessed its stability and accuracy through case-deletion and nonparametric bootstrap methods, implemented in the “bootnet” software package. We performed 1,000 bootstrap iterations per node to ensure reliable estimates (Epskamp et al., 2018). To evaluate edge accuracy, we calculated the 95% confidence intervals (CIs) for bootstrap edge weights; narrower CIs indicate greater accuracy (Su et al., 2025). We also computed correlation stability coefficients (CS coefficients) for the following centrality measures: strength, closeness, betweenness, expected influence, bridge strength, and bridge expected influence. The CS coefficient indicates the maximum proportion of cases that can be dropped while retaining, with 95% probability, a correlation ≥ 0.7 between the original centrality index and the centrality derived from the subset network (Epskamp et al., 2018). A CS value > 0.25 suggests acceptable stability, and a value > 0.5 indicates strong stability of the network (Jiang et al., 2025).

## 3 Results

### 3.1 Descriptive statistics

The study included 836 clinical nurses, comprising 46 males and 790 females. The mean age was (35.79 ± 6.51) years, and the mean years of service was (13.17 ± 7.26) years. Additional demographic characteristics are presented in Table 1.

### 3.2 Descriptive statistics of transformational leadership

The total transformational leadership score was (110.17 ± 12.53). The morale modeling dimension had the highest score among the four dimensions (34.06 ± 4.26). Table 2 presents the detailed scores for each dimension.

**Table 2:** Descriptive statistics of transformational leadership of clinical nurses (*N* = 836).

Variable	Dimensions	Number of items	Mean ± SD	
			Total mean score	Item mean score
Transformational leadership		26	110.17 ± 12.53	4.24 ± 0.48
	Morale modeling	8	34.06 ± 4.26	4.26 ± 0.53
	Inspirational motivation	6	25.49 ± 3.05	4.25 ± 0.51
	Individual consideration	6	25.09 ± 3.55	4.18 ± 0.59
	Charisma	6	25.54 ± 3.06	4.26 ± 0.51

### 3.3 Network structure

#### 3.3.1 Edge characteristics

**Figure 1:**
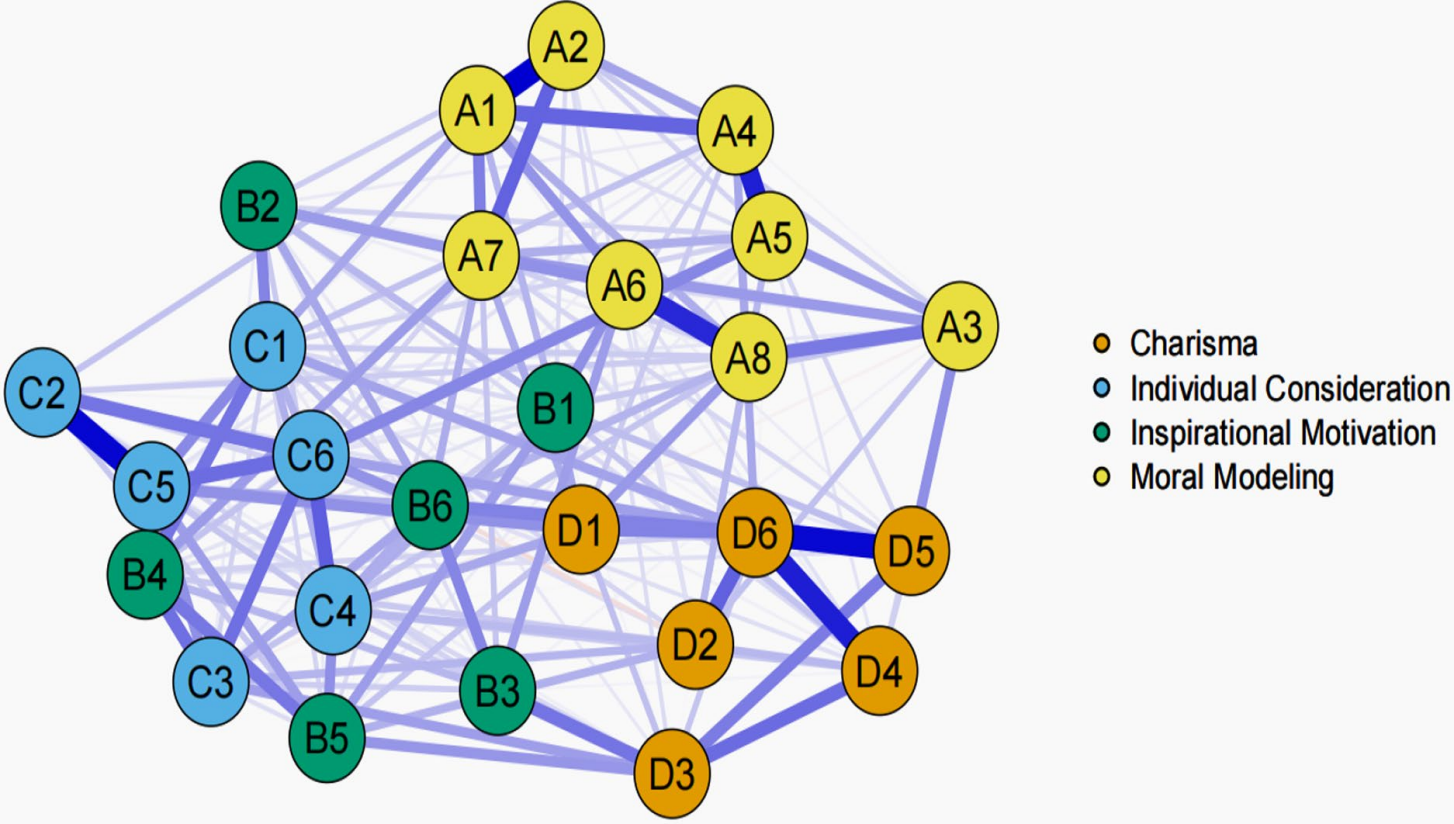
Network of items of transformational leadership the edge colour of blue indicates that the connection was positive, and red indicates that it was negative. The edge thickness and saturation indicate connection strength. The stronger the connection, the thicker the edge.

Figure 1 illustrates the network structure of internal relationships among transformational leadership components, which comprises 26 variables. Of the 325 possible edges, 195 (60.0%) were non-zero, indicating strong interconnectivity among the transformational leadership components. The strongest connection in the network was between A1 and A2 (edge weight = 0.265). This was followed by the connections between C2 and C5 (edge weight = 0.258), and between D5 and D6 (edge weight = 0.256).

Among inter-community edges, A1 was positively correlated with four nodes: B1, C1, C2, and C4; the strongest connection was to C1, with an edge weight of 0.087. A2 showed positive correlations with five inter-community nodes: B1, B2, C1, D2, and D3. The strongest edge weight (0.046) was associated with B2. A3 was connected to nine nodes: B1, B2, B3, C2, C3, C5, D2, D4, and D5, among which the edge to D2 had the strongest weight (0.059). A4 exhibited positive correlations with nine nodes: B1, B2, B4, B6, C1, C3, C5, D1, and D6. The strongest edge weight (0.086) was observed for the connection to D6. A5 was positively correlated with 11 nodes: B2, B5, B6, C1, C4, C6, D2, D3, D4, D5, and D6. The strongest edge weight (0.122) corresponded to its connection with C6. A6 showed positive correlations with six nodes: B1, B2, B3, D2, D4, and D5. The link to B1 had the strongest edge weight (0.112). A7 was positively correlated with 11 nodes: B2, B3, B4, B5, B6, C5, C6, D1, D4, D5, and D6. The strongest edge weight (0.100) was associated with B2. A8 exhibited positive correlations with eight nodes: B2, B6, C1, C2, C3, C5, D1, and D2. The strongest connection was to D1, with an edge weight of 0.102.

The moral modeling community contained 23 edges, with correlation values ranging from 0.011 to 0.265. The strongest correlation was between A1 and A2. The inspirational motivation community comprised 12 edges, with correlation values spanning 0.004–0.138. The strongest correlation occurred between B4 and B5. The individual consideration community contained 12 edges, with correlations ranging from 0.010 to 0.258. The strongest correlation was between C2 and C5. The charisma community included 12 edges, with correlation values between 0.044 and 0.256. The strongest correlation was between D5 and D6. Detailed correlations between all nodes in the network are provided in Supplementary Table 1.

#### 3.3.2 Nodes characteristics

**Figure 2:**
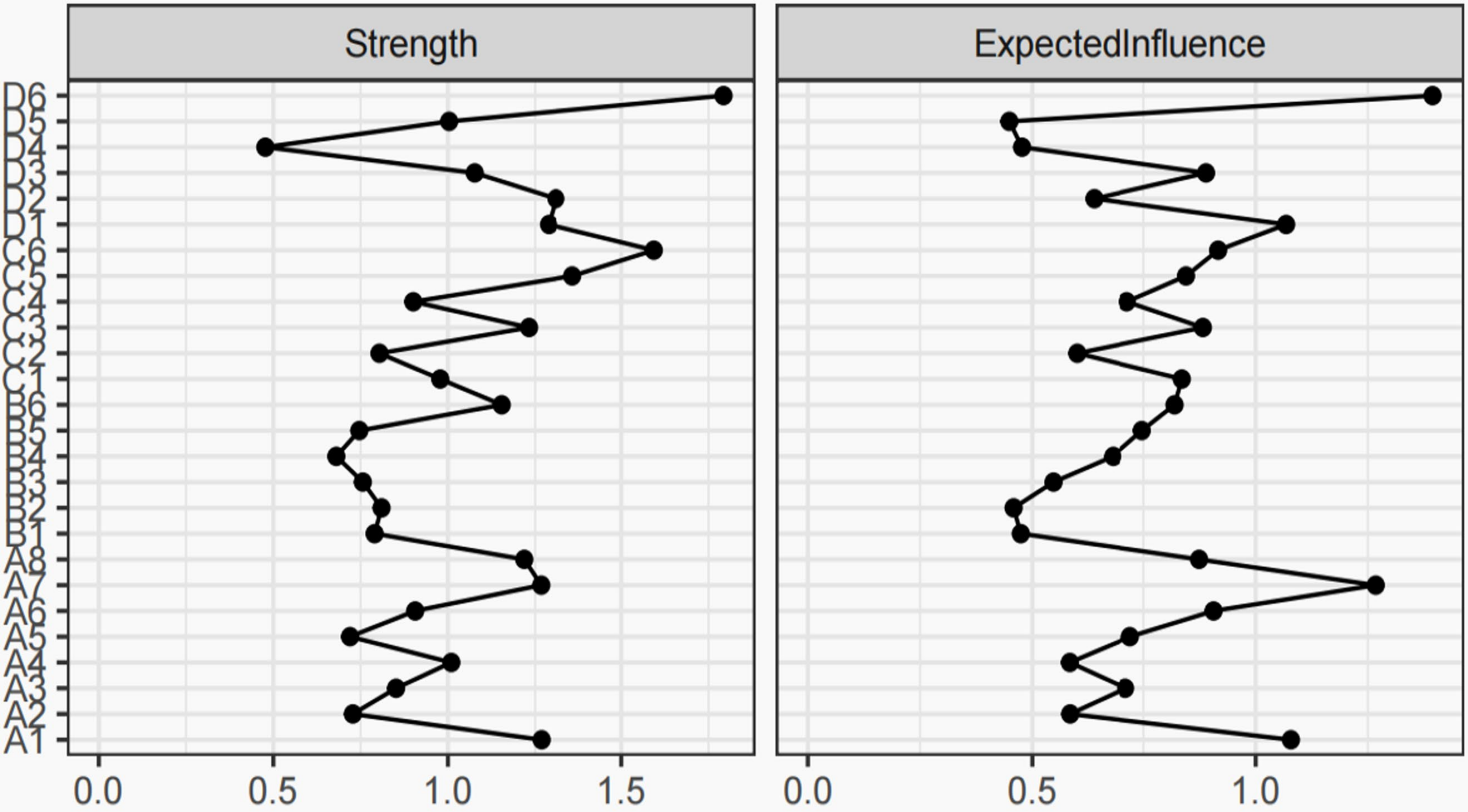
Strength and expected Influence of transformational leadership items in the present network.

Analysis of network centrality indices revealed that node D6 exhibited the highest strength centrality and expected influence, identifying it as the most influential element in the model. Nodes A7, C5, and C6 also showed relatively high strength and expected influence values, indicating their key roles in the model. In contrast, nodes B1, B5, C4, and D5 exhibited lower strength and expected influence values. This suggested that these nodes were the least connected within the network and played peripheral roles. Detailed centrality metrics for all nodes are presented in Table 3 and Figure 2.

**Table 3:** The characteristics and abbreviations for each item on transformational leadership scale.

Dimensions/Items	Abbreviation	Strength	Expected influence	Bridge Strength	Bridge Expected Influence
Moral modeling					
Uphold integrity, serve the public interest, and be devoid of personal gain	A1	0.36	0.40	0.23	0.23
Demonstrate resilience by undertaking hardships first and deferring personal benefits	A2	−0.97	−0.93	0.15	0.15
Prioritize collective goals over personal interests and dedicate full effort to work	A3	−0.90	−0.97	0.32	0.30
Willingly sacrifice personal interests for the benefit of the organization	A4	−0.57	−0.53	0.31	0.31
Consistently place collective and others’ interests above individual interests	A5	−0.43	−0.40	0.37	0.37
Never appropriate others’ work as your own	A6	−0.57	−0.53	0.36	0.36
Be willing to share both hardships and successes with the team	A7	1.68	1.72	0.60	0.60
Do not create undue difficulties for nursing staff or engage in retaliatory actions	A8	0.36	0.40	0.42	0.42
Inspirational motivation					
Facilitate an understanding of the hospital and department’s developmental prospects	B1	−0.01	0.02	0.69	0.69
Communicate clearly the operational philosophy and developmental goals of the hospital and department	B2	−1.11	−1.07	0.56	0.56
Articulate the long-term significance of your work	B3	−1.21	−1.17	0.48	0.48
Portray an aspirational future for the team	B4	−0.31	−0.27	0.62	0.62
Provide clear guidance toward defined goals and directions	B5	−0.20	−0.17	0.64	0.64
Regularly collaborate with the team to analyze how your work aligns with and impacts departmental and hospital objectives	B6	1.22	0.90	0.86	0.80
Individual consideration					
Consider individual circumstances when interacting with team members	C1	1.09	1.13	0.81	0.81
Be willing to assist with team members’ personal and family challenges	C2	−1.19	−1.15	0.32	0.32
Maintain open communication to understand team members’ professional, personal, and family situations	C3	−0.38	−0.45	0.57	0.55
Provide patient mentorship and resolve questions from team members	C4	−0.23	−0.20	0.59	0.59
Show concern for team members’ professional work, personal wellbeing, and career growth, and offer sincere developmental advice	C5	1.67	1.70	0.54	0.54
Focus on creating opportunities for team members to leverage their strengths	C6	1.55	1.58	0.53	0.53
Charisma					
Possess exceptional professional competence	D1	−0.26	−0.23	0.65	0.65
Exhibit open-mindedness and strong innovative thinking	D2	−0.54	−0.86	0.61	0.55
Be passionate about your work and demonstrate strong ambition and drive	D3	−0.53	−0.49	0.45	0.45
Be deeply committed to your duties and maintain consistent enthusiasm	D4	−0.69	−0.66	0.32	0.32
Engage in continuous learning to enhance your capabilities	D5	−0.26	−0.22	0.41	0.41
Demonstrate decisiveness and assertiveness in management, and be adept at resolving complex issues	D6	2.42	2.45	0.53	0.53

**Figure 3:**
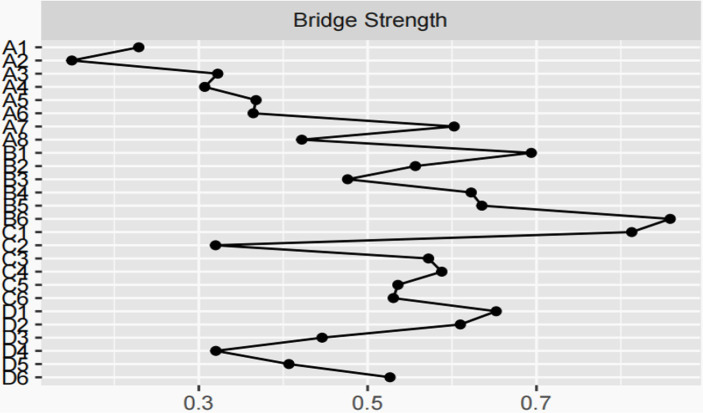
Bridge Strength of transformational leadership items in the present network.

**Figure 4:**
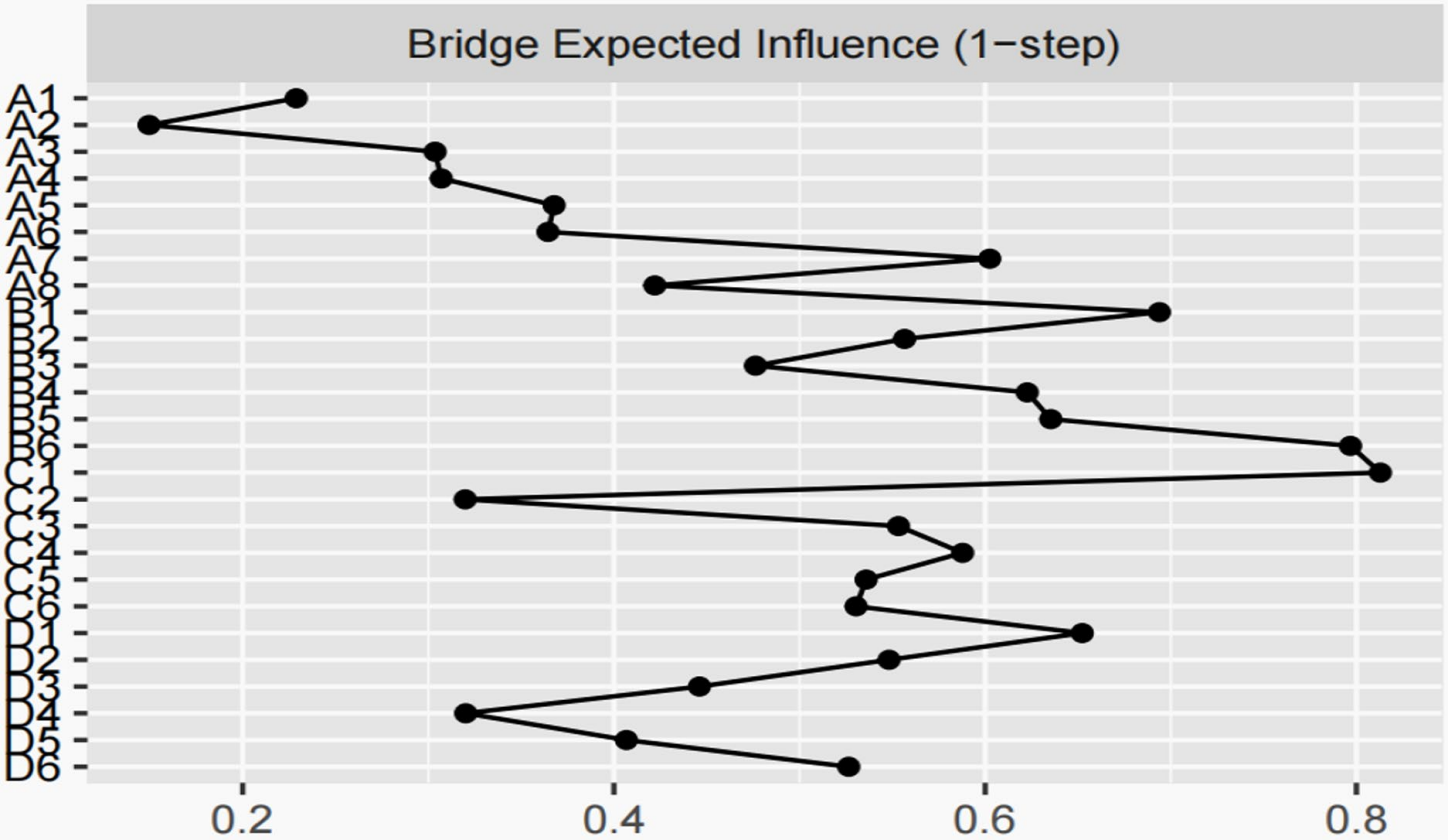
Bridge expected influence (1-step) of transformational leadership items in the present network.

**Figure 5:**
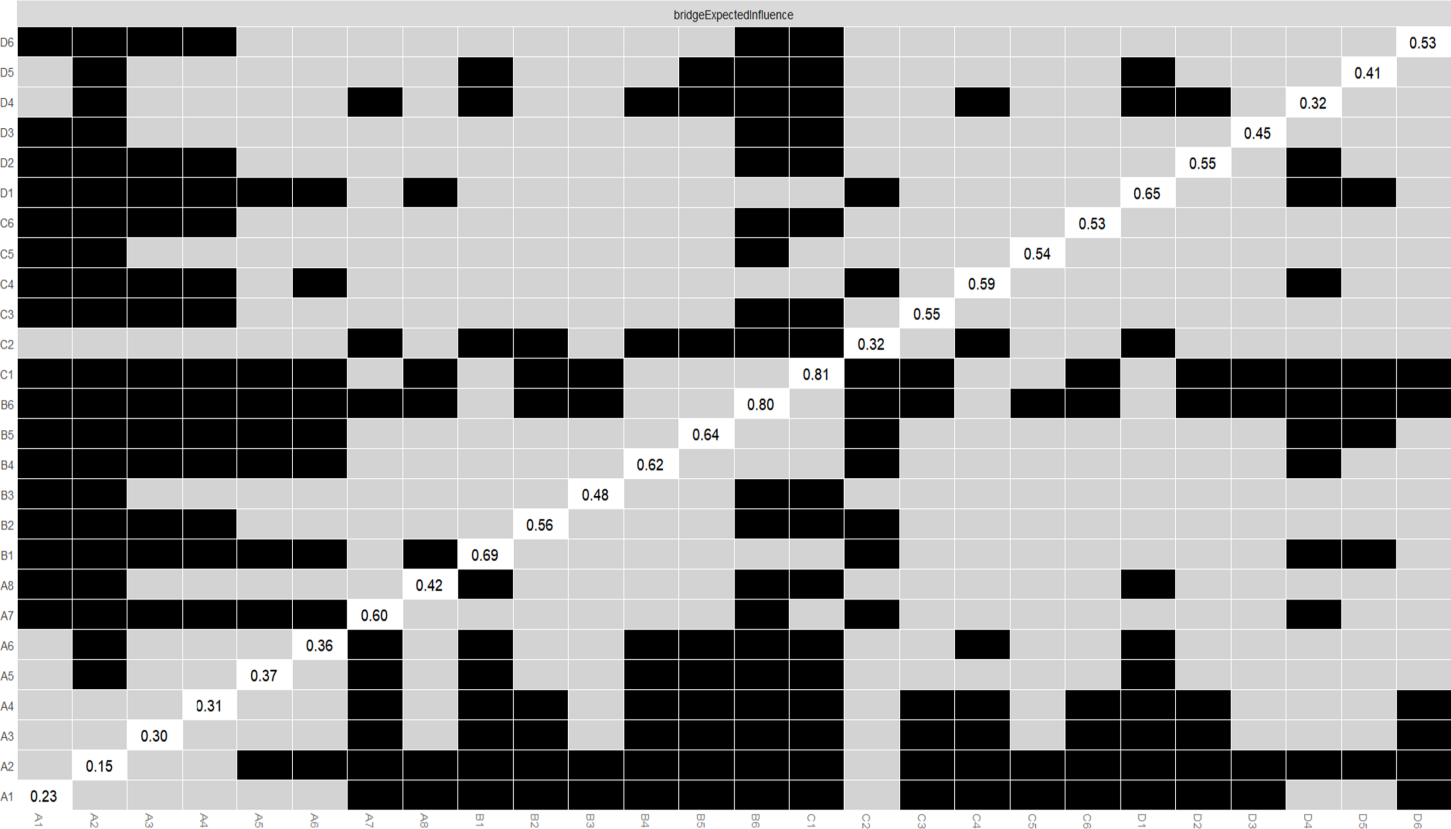
Bootstrapped difference test for node bridge expected influences gray boxes indicate node bridge expected influences that do not differ significantly from one another, while black boxes indicate node bridge expected influences that do differ significantly.

In terms of bridge centrality, the nodes with the highest bridge strength and expected influence were B6, C1, B1, and D1. This indicated that these nodes played a critical bridging role within the transformational leadership network. Among the four communities of transformational leadership, the following nodes demonstrated the highest bridge expected influence within their respective communities: A7, B6, C1, and D1. These nodes acted as core connectors that linked the other three communities. In contrast, A2 showed the lowest bridge strength and bridge expected influence across the entire transformational leadership network. Its cross-group connectivity was relatively weak, which limited its role in facilitating overall network coordination. The detailed bridge centrality characteristics of each node are presented in Figures 3–5.

#### 3.3.3 Network stability and accuracy

**Figure 6:**
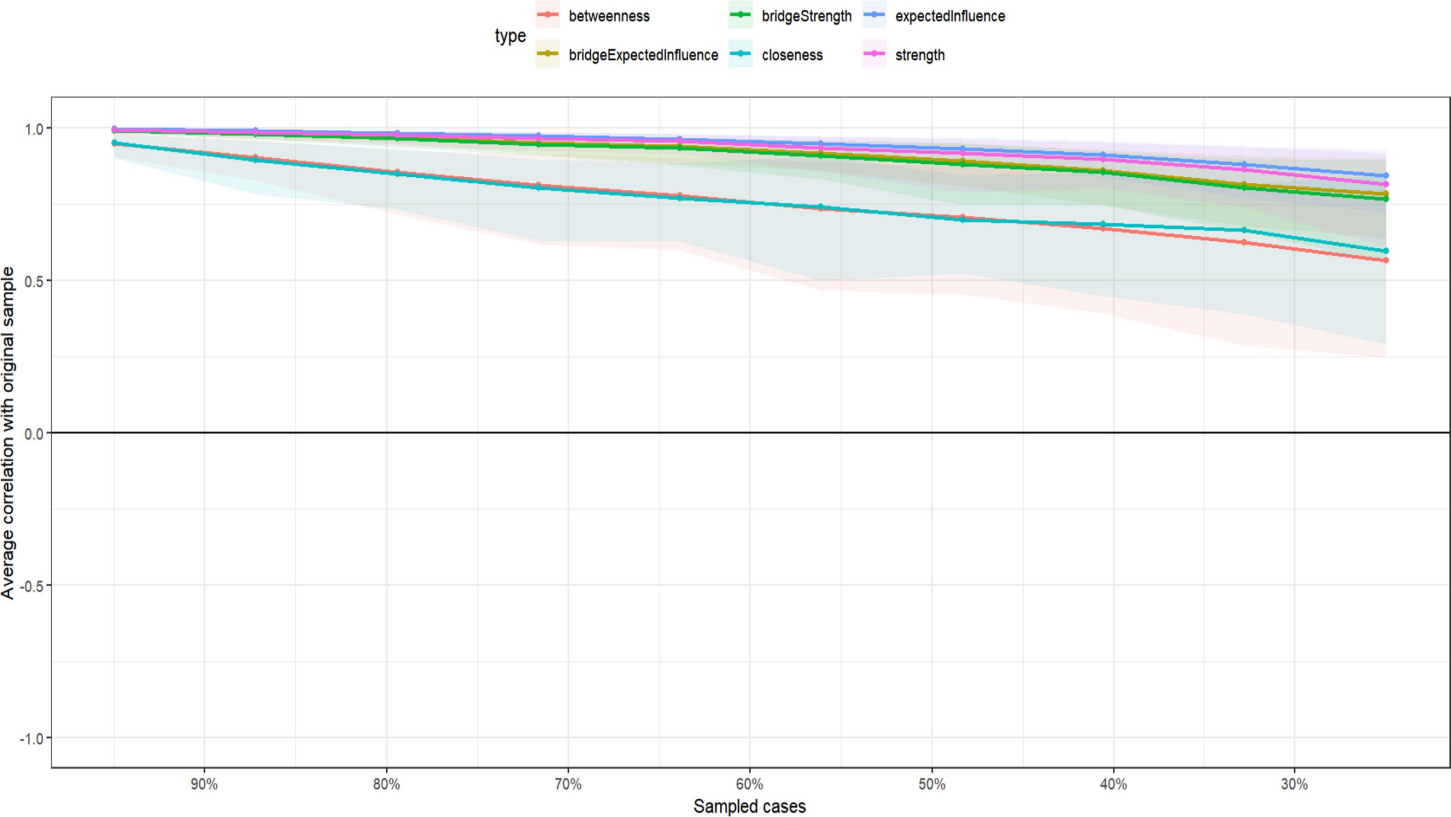
Stability of node centrality and bridge centrality.

**Figure 7:**
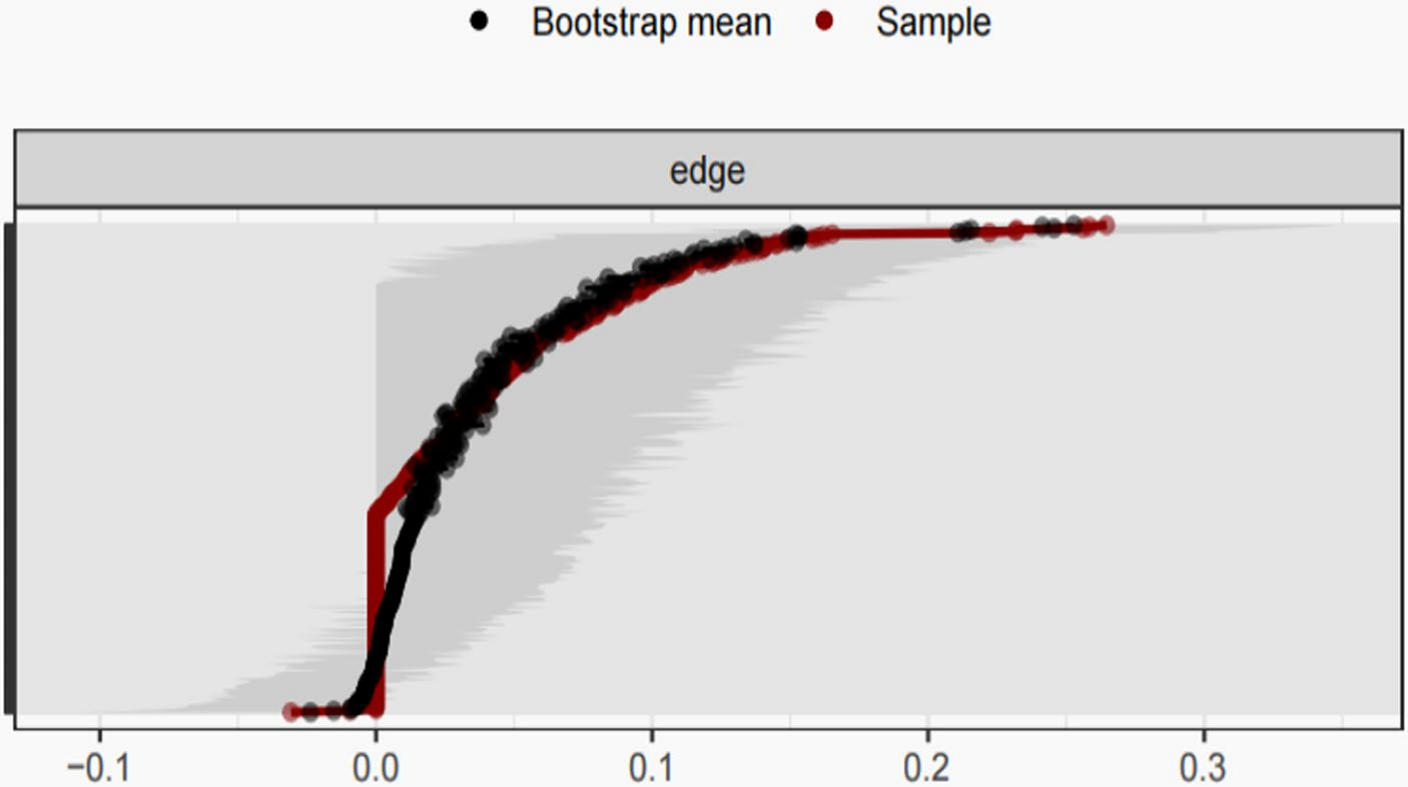
Accuracy of edge weights the red line depicts the sample edge weights and the gray bar depicts the bootstrapped confidence interval.

**Figure 8:**
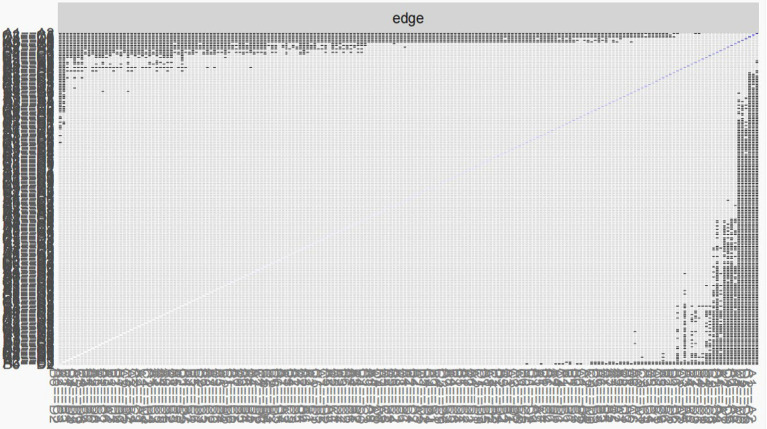
Bootstrapped difference test for edge weights gray boxes indicate edge weights that do not differ significantly from one another, while black boxes indicate edge weights that do differ significantly. Blue and red boxes on the diagonal correspond to edge weights with positive and negative correlations, respectively.

Results are presented in Figure 6. The CS coefficients for betweenness and closeness were 0.206 and 0.283, respectively, both below the 0.5 threshold. In contrast, CS coefficients for strength, expected influence, bridge strength, and bridge expected influence were 0.672, 0.75, 0.594, and 0.672, respectively, all exceeding 0.5. This indicated that the network structure remained largely unchanged after omitting 75% of the sample, demonstrating strong stability. Additionally, the edge weights estimated via nonparametric bootstrap generally had narrow 95% confidence intervals (Figure 7), indicating high accuracy in network estimation. Furthermore, the edge weight significance test showed that the strongest cross-community connection was between B4 and C3, as shown in Figure 8.

## 4 Discussion

The four dimensions of transformational leadership do not exist in isolation; rather, they form an organic whole through interconnections among their respective items. In the network model constructed in this study, 60.0% of non-zero edges demonstrate the close interrelationships among these dimensions and items. Furthermore, significant interactions exist both within and across dimensions, which aligns closely with the core theoretical essence of transformational leadership, “comprehensive empowerment and multidimensional synergy.”

### 4.1 Transformational leadership scores: characteristic features and core dimensions reveal their value

The total transformational leadership score in this study was (110.17 ± 12.53), which is slightly higher than that reported by Wu et al. (2025a) in their survey of 590 ICU nurses from six tertiary hospitals in China. This discrepancy primarily stems from the high-risk, high-workload nature of ICU settings, which leads to differences in management approaches, communication patterns, psychosocial and environmental factors, team structures, and professional development pathways (Huang et al., 2025). Additionally, no statistically significant differences were observed in perceived transformational leadership ratings across nurses of different professional titles. This finding can be explained by two factors. First, self-report scales are susceptible to social desirability bias. Respondents tend to portray leadership behaviors favorably and downplay negative aspects, thereby aligning assessment results with socially idealized standards. Second, the sample for this study consisted entirely of nurses from Grade A tertiary hospitals, where standardized nursing management training systems and uniform leadership assessment criteria have been established, thereby reducing the scoring gap across different professional titles (International Council of Nurses, 2019; Zhang et al., 2021). Among the four dimensions, the moral modeling dimension received the highest score. This finding is consistent with results from a self-administered questionnaire study by Wu et al. (2020) on spiritual atmosphere, emotional exhaustion, clinical leadership, and turnover intention among nurses in two hospitals in Jiangsu Province. Transformational leaders act as role models. By adopting this leadership style, nursing managers inspire team cohesion and enhance nurses’ professional identity through their expertise and sense of accountability, thereby promoting continuous improvement in nursing care quality (Ding and Cao, 2024). The individual consideration dimension received the highest total score, suggesting its potential contribution to overall leadership effectiveness within the transformational leadership framework. Nursing is widely recognized as one of the most stressful occupations compared to other professions (Patrician et al., 2022). Personalized care from leaders can stimulate nurses’ intrinsic motivation, encouraging more active engagement in professional development and patient care. This enables nurses to maintain a high level of professional efficacy even under high-workload conditions (Labrague and Obeidat, 2022). This finding aligns with the nursing management philosophy in China, which emphasizes humanistic care and team cohesion (Wang et al., 2024).

### 4.2 The core supporting functions of key nodes and bridge variables in the network

The research findings indicate that D6, A7, C6, and C5 exhibit the highest strength and expected influence. These values represent statistical centrality indices calculated from the network structure, reflecting their relative importance within the observed associations. This outcome results from the combined influence of China’s clinical nursing organizational context and cultural characteristics, representing a significant contribution to the existing literature. In China’s clinical nursing organizational context, the post-pandemic era is characterized by nursing staff shortages and workload surges. Clinical nursing management frequently faces thorny issues, including doctor-patient communication conflicts, interdepartmental collaboration challenges, and sudden emergencies (Wang et al., 2024). As a core node of the intellectual stimulation dimension, D6 embodies “bold leadership and problem-solving ability” traits that are aligned with the core needs of current nursing managers in addressing complex management scenarios and resolving dilemmas. These characteristics were positively associated with management efficiency and team stress levels. From a cultural perspective, nursing teams in China emphasize “unity of purpose and collaborative action” (Gu et al., 2026). The trait “sharing hardships and joys” embodied by A7 aligns with the core concept of “mutual assistance and collaboration” in collectivist culture. It could bridge the psychological gap between managers and nurses and strengthen team cohesion. This aligns with the professional characteristics of Chinese nursing teams (“strong cohesion and a focus on collaboration”). As key nodes in the personalized care dimension, C5 and C6 derive their central importance from the current state and humanistic orientation of clinical nursing in China. Currently, the incidence of occupational burnout among nurses in China remains high, and retention challenges persist (Ren et al., 2023). Nurses face not only intense work pressure but also significant psychological stress. Items C5 (“Caring for work-life balance and professional growth”) and C6 (“Creating conditions to leverage strengths”) align with nurses’ dual needs for humanistic care and professional development. The observed associations suggest that addressing these needs may be important for psychological stress management, professional belonging, and professional growth through empowerment. These findings are consistent with the humanistic orientation of nursing management in China (Yuan et al., 2022) and extend prior work on key nodes within China’s nursing humanistic context, which remained underexplored.

In addition to variables with high strength and expected influence, those with high bridge strength and bridge expected influence also warrant attention. Bridge centrality clarifies the distinct roles nodes play in connecting different network communities (Wu C. et al., 2025). Here, C1, B6, B1, and D1 show the highest bridge strength and bridge expected influence. This finding overcomes a limitation of previous studies—which focused solely on individual dimensions—and clarifies the mechanism of synergistic interaction among the theoretical dimensions of transformational leadership. As bridge nodes statistically connecting the four dimensions, these items demonstrate associations with the inter-relationships among idealized influence, inspirational motivation, personalized consideration, and intellectual stimulation, highlighting potential synergistic patterns within this context. The findings extend the transformational leadership framework by highlighting the network-based perspective on multidimensional synergy within this context and offer new insights into the integration of leadership dimensions in Chinese nursing settings.

### 4.3 Significant internal correlations within each dimension demonstrate the inherent logic of the leadership framework

In the moral modeling community, the strongest correlation was observed between A4 and A5. Additionally, two further strong associations were identified: between A6 and A8, and between A1 and A2. These results are consistent with a cross-sectional study of 297 nurses at Mansoura University Hospital in Egypt (AbdELhay et al., 2025), which indicated that these elements are closely linked within the survey structure, which may suggest potential areas for enhancing workplace culture, though their direct impact on specific practical outcomes requires further investigation. This collective-interest-oriented leadership behavior substantially strengthens team cohesion and organizational identification. The nursing profession in China is deeply influenced by a collectivist culture (Jin et al., 2019), with “selfless dedication and prioritizing the collective good” as its core values (Felfe et al., 2008). This cultural orientation, as manifested in nursing management, aligns closely with the “people-oriented, work-dedicated” organizational culture of Chinese medical institutions. Within the inspirational motivation community, the strongest association occurred between B4 and B5. This pattern suggests a close relationship between these elements in the measurement framework. Existing research indicates that transformational leadership may foster motivating work environments, as empowering, supporting, and inspiring nurses through such leadership has been associated with reduced burnout and enhanced job satisfaction and retention rates (AbdELhay et al., 2025). However, the strength of this relationship and its direct causal impact warrant further investigation beyond the observed correlations. Within the individual consideration community, C2 and C5 demonstrated the strongest linkage. In this study, 77% of the nurses were married and managed dual roles in family caregiving and clinical work, creating a strong need for work-life balance. This supports key research concluding that healthcare organizations should address nurses’ work–life issues to improve their overall wellbeing (Gribben and Semple, 2021). A study of 656 nurses in Dhaka, Bangladesh, by Rony et al. (2023) on determinants of work–life balance also suggests that supporting work–life balance may be important for improving healthcare productivity, patient care, and clinical outcomes. In the charisma community, the strongest correlations were between D5 and D6. Nursing is a growth-oriented profession. The increasing demand for high-quality care in clinical settings reflects the social value placed on nurses, which motivates them to engage in continuous learning to improve their professional competence and academic qualifications (Zhang et al., 2024).

### 4.4 Transformational leadership meets the operational demands of the nursing profession

Given the global shortage of nursing professionals, it is crucial to design and implement strategies that safeguard nurses’ wellbeing and strengthen their professional commitment. Investing in positive and effective leadership models is one of the most impactful and feasible measures in this field (Montenegro Méndez et al., 2025). At the same time, implementing work-life balance initiatives is essential to enhance staff retention and reduce emotional exhaustion. Nurses’ organizational commitment is a core factor influencing their retention. Previous studies have shown an association between transformational leadership and nurses’ organizational commitment (Haoyan et al., 2023).

With the evolution of modern nursing management in China, nursing leadership is transitioning from a paternalistic to a transformational style. This forward-looking and proactive approach shifts the focus from rigid discipline to empowering nurses and promoting their participation. Research suggests that transformational leadership is associated with addressing higher-level needs, mobilizing intrinsic motivation, enhancing team cohesion and belonging, and is linked to improvements in work drive and creativity among nurses (Wu et al., 2025b).

### 4.5 Recommendations for leadership development pathways based on research findings

Therefore, healthcare institutions should prioritize critical development programs for transformational leadership. Research findings suggest that this approach may be associated with a more precise and efficient enhancement of nursing managers’ transformational leadership capabilities, which is correlated with their autonomy in nursing practice (Omidvar et al., 2025). First, to cultivate and promote a transformational leadership style, leaders ought to respond to nurses’ needs and emotions by actively soliciting their opinions and suggestions; they should also motivate and encourage nurses to foster their enthusiasm and innovative potential, helping them perceive the meaning and value of their work; moreover, establish a positive, open, and inclusive work environment that encourages nurses’ participation in decision-making, thereby promoting their sense of belonging and professional responsibility (Wu et al., 2025a). Additionally, implementing a performance evaluation system focused on transformational leadership behaviors may be associated with advanced practices that support professional autonomy in clinical environments (Omidvar et al., 2025).

Transformational leadership research spans multiple disciplines (Chen et al., 2026; Flokou et al., 2025; Peng and Wong, 2025), and the practical elements across its dimensions are interrelated. Using network analysis, this study explores potential pathways for enhancing transformational leadership from multiple perspectives, thereby contributing insights to address gaps in the existing literature.

## 5 Conclusion

This study reveals the rich and complex relationships embedded in the transformational leadership framework. From a network analysis perspective, D6, A7, C5, and C6 demonstrate higher centrality metrics in the current network. Meanwhile, C1, B6, B1, and D1 exhibit elevated bridge centrality measures, reflecting their statistically significant connections across different communities.

These findings provide important guidance for clinical nursing practice and offer potential intervention pathways and practical references for enhancing transformational leadership among nurse managers. For future research, this study suggests the following: On the one hand, efforts may focus on strengthening competencies associated with the four key nodes (D6, A7, C5, and C6) by conducting targeted training programs on “handling difficult issues,” “team collaboration,” “humanistic care,” and “professional empowerment”—in alignment with the cultural and organizational needs of nursing management in China. However, it should be noted that network centrality indices do not necessarily indicate priority for intervention planning. On the other hand, the role of bridge nodes such as C1, B6, B1, and D1 may warrant further investigation. Network-derived bridge functions are statistical concepts that do not automatically translate to enhanced leadership performance in practice. Future empirical studies are needed to validate whether strengthening the competencies associated with these nodes could potentially contribute to the four dimensions of transformational leadership in practice. Additionally, these key nodes could be considered for incorporation into the performance evaluation system for nursing managers as one possible reference, to guide them in actively practicing relevant behaviors. Longitudinal research is required to determine whether such approaches might further optimize the nursing practice environment, mitigate nurse burnout, reduce turnover rates, and support continuous improvement of nursing management standards in China.

### 5.1 Implications and significance

This study’s findings offer preliminary theoretical insights and practical guidance for research on transformational leadership, clinical nursing practice, and healthcare institution management.

#### 5.1.1 At the theoretical level

This study complements and enriches scholarly understanding of the structural relationships among the 26 items of the Transformational Leadership Scale. Network analysis results indicate that the items on the scale are not entirely independent but are interrelated. Additionally, there is a certain degree of correlation among the scale’s higher-order dimensions. The findings of this study provide preliminary empirical evidence for research on the structure of the Transformational Leadership Scale. Notably, our dataset revealed heterogeneous association strengths among items. These findings, specific to this survey, suggest that the uniform weighting approach may warrant consideration within the context of the observed data characteristics. Whether the current scoring framework performs optimally in similar survey contexts merits further empirical exploration.

#### 5.1.2 At the practical level

Some high-impact core items identified in this study may serve as potential targets for enhancing nursing managers’ transformational leadership capabilities. Conducting targeted training on core nodes such as D6, A7, C5, and C6 may further bolster these capabilities. In clinical practice, nursing managers can foster a motivating work environment and implement management strategies, including personalized care and intellectual stimulation for frontline nurses. These measures could be associated with reduced nurses’ occupational stress and burnout while increasing their sense of professional belonging. To a certain extent, the optimized transformational leadership management model may be associated with improvements in the quality of clinical nursing services. It is also expected to achieve a win-win outcome for nursing managers, frontline nurses, and patients. However, the feasibility and effectiveness of the aforementioned targeted intervention strategies require further validation through longitudinal or intervention studies.

#### 5.1.3 At the organizational management level

Healthcare institutions may refer to this study’s findings on mediating centrality. Based on key mediating nodes such as C1, B6, B1, and D1, they can appropriately optimize the design and implementation of nursing leadership training programs. Strengthening resource integration, work coordination, and collaborative linkages among these key intermediary nodes may help maintain the integrity and stable operational efficiency of the nursing service chain under resource-constrained conditions. This may address common resource-related issues such as declining nursing quality, disruptions in care continuity, and insufficient service accessibility. Additionally, nursing managers should continuously enhance their overall competence and demonstrate transformational leadership traits. By clarifying career development visions for frontline nurses and implementing reasonable empowerment management strategies, nursing managers may stimulate nurses’ professional potential, which could facilitate the synergistic development of individual and organizational goals.

Given the increasingly acute global nursing shortage, the transformational leadership optimization strategy proposed in this study may help refine clinical nursing management models, enhance the resilience of the nursing workforce, and promote the sustainable provision and continuous quality improvement of nursing services. Nevertheless, its practical value and generalizability require further empirical testing across different organizational contexts.

### 5.2 Limitations

This study has several limitations that warrant careful consideration. First, the cross-sectional design of this study precludes causal inferences and can only establish associations among variables. Second, data were collected via convenience sampling of clinical nurses from five provinces and municipalities using the QuestionStar platform. Although this cross-regional, multi-site convenience sampling increased geographic coverage, it remains prone to selection bias and unmeasured clustering effects. Consequently, the findings may not fully generalize to the broader population of clinical nurses. Differences in management models and nursing cultures across hospitals may introduce sample heterogeneity, which could affect the strength of network node connections and the accuracy of central node identification, thereby limiting the generalizability of the findings. Third, the results are specific to the Chinese nursing context; replication in other cultural and healthcare settings is needed to establish the cross-cultural validity of the network structure. Additionally, this study did not include outcome variables (e.g., nurse burnout, turnover rates) to validate the clinical impact of the identified key nodes. At the same time, there is a significant gender imbalance among the study participants. Future research should therefore include more male nurses to balance the sample’s gender composition. Longitudinal studies that incorporate clinical outcome measures would strengthen the evidence for the practical significance of the network findings. Subsequent research could expand the sample size and conduct further multivariate analyses.

## Data Availability

The data analyzed in this study is subject to the following licenses/restrictions: no datasets were generated or analyzed during the current study. Requests to access these datasets should be directed to NZ 1241332783@qq.com.
