# Supplementary material for: The internal structure of transformational leadership as perceived by clinical nurses: a network analysis
Source: Front Psychol. 2026 Jul 29;17:1878851. doi: 10.3389/fpsyg.2026.1878851 (PMC13461476; doi:10.3389/fpsyg.2026.1878851)
Supplement: Supplementary file 1 [file Table_1.docx]

**Table 1** The correlation matrix in the network of each community

|  | **A1** | **A2** | **A3** | **A4** | **A5** | **A6** | **A7** | **A8** | **B1** | **B2** | **B3** | **B4** | **B5** | **B6** | **C1** | **C2** | **C3** | **C4** | **C5** | **C6** | **D1** | **D2** | **D3** | **D4** | **D5** | **D6** |
| --- | --- | --- | --- | --- | --- | --- | --- | --- | --- | --- | --- | --- | --- | --- | --- | --- | --- | --- | --- | --- | --- | --- | --- | --- | --- | --- |
| **A1** | **0.000** |  |  |  |  |  |  |  |  |  |  |  |  |  |  |  |  |  |  |  |  |  |  |  |  |  |
| **A2** | **0.265** | **0.000** |  |  |  |  |  |  |  |  |  |  |  |  |  |  |  |  |  |  |  |  |  |  |  |  |
| **A3** | **0.030** | **0.051** | **0.000** |  |  |  |  |  |  |  |  |  |  |  |  |  |  |  |  |  |  |  |  |  |  |  |
| **A4** | **0.158** | **0.091** | **0.006** | **0.000** |  |  |  |  |  |  |  |  |  |  |  |  |  |  |  |  |  |  |  |  |  |  |
| **A5** | **0.000** | **0.039** | **0.099** | **0.232** | **0.000** |  |  |  |  |  |  |  |  |  |  |  |  |  |  |  |  |  |  |  |  |  |
| **A6** | **0.107** | **0.000** | **0.033** | **0.000** | **0.000** | **0.000** |  |  |  |  |  |  |  |  |  |  |  |  |  |  |  |  |  |  |  |  |
| **A7** | **0.130** | **0.160** | **0.101** | **0.000** | **0.080** | **0.110** | **0.000** |  |  |  |  |  |  |  |  |  |  |  |  |  |  |  |  |  |  |  |
| **A8** | **0.080** | **0.011** | **0.135** | **0.043** | **0.042** | **0.222** | **0.045** | **0.000** |  |  |  |  |  |  |  |  |  |  |  |  |  |  |  |  |  |  |
| **B1** | **0.072** | **0.034** | **0.054** | **0.041** | **0.000** | **0.112** | **0.000** | **0.000** | **0.000** |  |  |  |  |  |  |  |  |  |  |  |  |  |  |  |  |  |
| **B2** | **0.000** | **0.046** | **0.009** | **0.016** | **0.042** | **0.090** | **0.100** | **0.013** | **0.069** | **0.000** |  |  |  |  |  |  |  |  |  |  |  |  |  |  |  |  |
| **B3** | **0.000** | **0.000** | **0.008** | **0.000** | **0.000** | **0.097** | **0.055** | **0.000** | **0.000** | **0.029** | **0.000** |  |  |  |  |  |  |  |  |  |  |  |  |  |  |  |
| **B4** | **0.000** | **0.000** | **0.000** | **0.026** | **0.000** | **0.000** | **0.098** | **0.000** | **0.024** | **0.000** | **0.070** | **0.000** |  |  |  |  |  |  |  |  |  |  |  |  |  |  |
| **B5** | **0.000** | **0.000** | **0.000** | **0.000** | **0.015** | **0.000** | **0.032** | **0.000** | **0.097** | **0.000** | **0.026** | **0.138** | **0.000** |  |  |  |  |  |  |  |  |  |  |  |  |  |
| **B6** | **0.000** | **0.000** | **0.000** | **0.004** | **0.006** | **0.000** | **0.081** | **0.074** | **0.048** | **0.087** | **0.123** | **0.028** | **0.004** | **0.000** |  |  |  |  |  |  |  |  |  |  |  |  |
| **C1** | **0.087** | **0.011** | **0.000** | **0.060** | **0.042** | **0.000** | **0.000** | **0.048** | **0.034** | **0.125** | **0.016** | **0.141** | **0.040** | **0.054** | **0.000** |  |  |  |  |  |  |  |  |  |  |  |
| **C2** | **0.058** | **0.000** | **0.019** | **0.000** | **0.000** | **0.000** | **0.000** | **0.048** | **0.005** | **0.000** | **0.040** | **0.000** | **0.053** | **0.041** | **0.000** | **0.000** |  |  |  |  |  |  |  |  |  |  |
| **C3** | **0.000** | **0.000** | **-0.009** | **0.012** | **0.000** | **0.000** | **0.000** | **0.056** | **0.000** | **0.015** | **0.046** | **0.145** | **0.030** | **0.088** | **0.042** | **0.000** | **0.000** |  |  |  |  |  |  |  |  |  |
| **C4** | **0.000** | **0.000** | **0.000** | **0.000** | **0.005** | **0.000** | **0.000** | **0.000** | **0.080** | **0.050** | **0.047** | **0.003** | **0.112** | **0.081** | **0.078** | **0.010** | **0.000** | **0.000** |  |  |  |  |  |  |  |  |
| **C5** | **0.000** | **0.000** | **0.012** | **0.012** | **0.000** | **0.000** | **0.025** | **0.019** | **0.015** | **0.000** | **0.020** | **0.031** | **0.096** | **0.075** | **0.118** | **0.258** | **0.111** | **0.055** | **0.000** |  |  |  |  |  |  |  |
| **C6** | **0.000** | **0.000** | **0.000** | **0.000** | **0.122** | **0.000** | **0.041** | **0.000** | **0.024** | **0.000** | **0.000** | **0.057** | **0.018** | **0.108** | **0.075** | **0.140** | **0.145** | **0.165** | **0.148** | **0.000** |  |  |  |  |  |  |
| **D1** | **0.000** | **0.000** | **0.000** | **0.049** | **0.000** | **0.000** | **0.083** | **0.102** | **0.088** | **0.000** | **0.000** | **0.000** | **0.057** | **0.059** | **0.000** | **0.018** | **0.000** | **0.086** | **0.110** | **0.000** | **0.000** |  |  |  |  |  |
| **D2** | **0.000** | **0.027** | **0.059** | **0.000** | **0.070** | **0.026** | **0.000** | **0.063** | **0.000** | **0.000** | **0.000** | **0.050** | **0.076** | **-0.031** | **0.038** | **0.000** | **0.072** | **0.068** | **0.000** | **0.029** | **0.000** | **0.000** |  |  |  |  |
| **D3** | **0.000** | **0.032** | **0.000** | **0.000** | **0.002** | **0.000** | **0.000** | **0.000** | **0.006** | **0.018** | **0.140** | **0.000** | **0.105** | **0.000** | **0.000** | **0.011** | **0.096** | **0.000** | **0.036** | **0.000** | **0.060** | **0.000** | **0.000** |  |  |  |
| **D4** | **0.012** | **0.000** | **0.040** | **0.000** | **0.022** | **0.026** | **0.030** | **0.000** | **0.057** | **0.033** | **0.007** | **0.001** | **0.000** | **0.000** | **0.011** | **0.000** | **0.000** | **0.054** | **0.000** | **0.028** | **0.000** | **0.070** | **0.149** | **0.000** |  |  |
| **D5** | **0.000** | **0.000** | **0.112** | **0.000** | **0.039** | **0.013** | **0.037** | **0.000** | **0.063** | **0.000** | **0.000** | **0.027** | **0.000** | **0.030** | **0.016** | **0.000** | **0.000** | **0.002** | **0.060** | **0.009** | **0.052** | **0.000** | **0.133** | **0.044** | **0.000** |  |
| **D6** | **0.000** | **0.000** | **0.000** | **0.086** | **0.005** | **0.000** | **0.020** | **0.000** | **0.008** | **0.000** | **0.000** | **0.042** | **0.000** | **0.126** | **0.091** | **0.027** | **0.002** | **0.000** | **0.024** | **0.095** | **0.125** | **0.162** | **0.055** | **0.232** | **0.256** | **0.000** |
|  | **A1** | **A2** | **A3** | **A4** | **A5** | **A6** | **A7** | **A8** | **B1** | **B2** | **B3** | **B4** | **B5** | **B6** | **C1** | **C2** | **C3** | **C4** | **C5** | **C6** | **D1** | **D2** | **D3** | **D4** | **D5** | **D6** |
